# Four novel polymorphisms in long non-coding RNA HOTTIP are associated with the risk and prognosis of colorectal cancer

**DOI:** 10.1042/BSR20180573

**Published:** 2019-05-07

**Authors:** Zhi Lv, Qian Xu, Liping Sun, Jing Wen, Xinxin Fang, Chengzhong Xing, Yuan Yuan

**Affiliations:** 1 Tumor Etiology and Screening Department of Cancer Institute and General Surgery, the First Hospital of China Medical University, Shenyang 110001, China; 2 Key Laboratory of Cancer Etiology and Prevention in Liaoning Education Department, the First Hospital of China Medical University, Shenyang 110001, China; 3 Key Laboratory of GI Cancer Etiology and Prevention in Liaoning Province, the First Hospital of China Medical University, Shenyang 110001, China

**Keywords:** colorectal cancer, HOTTIP, polymorphism, prognosis, susceptibility

## Abstract

Background: The role of long non-coding RNA (lncRNA) HOXA transcript at the distal tip (HOTTIP) as an oncogene in varieties of human cancer including colorectal cancer (CRC) has been extensively researched. The expression and function of lncRNAs could be affected by single nucleotide polymorphisms (SNPs), which are associated with cancer susceptibility and prognosis. However, no investigation has focused on the association between HOTTIP SNPs and CRC. The aim of the present study was to explore the association of polymorphisms in the lncRNA HOTTIP gene with CRC risk and prognosis. Methods: A total of 1848 subjects were enrolled in our study, including 884 CRC cases and 964 controls. Genotyping for five HOTTIP tagSNPs (rs3807598, rs17501292, rs2067087, rs17427960, and rs78248039) was performed by applying Kompetitive allele specific PCR (KASP). Results: The results showed three SNPs (rs3807598, rs2067087, and rs17427960) were associated with enhanced CRC risk both in overall and stratified analysis. One polymorphism, rs17501292, could improve the overall survival (OS) of CRC patients in the tumor of ulcerative/invasive-type subgroup. Conclusion: These findings suggest HOTTIP SNPs could potentially be predictive biomarkers for CRC risk and prognosis. The present study provides clues for further exploration of novel lncRNA-based genetic biomarkers to predict CRC susceptibility as well as clinical outcome.

## Introduction

Colorectal cancer (CRC) is the third most common malignancy and the fourth most frequent cause of cancer-related deaths worldwide [1,2]. Similar to the majority of other malignancies, a lack of genetic markers for tumorigenesis and development remains one of the most critical obstacles challenging CRC diagnosis and therapy [3]. Therefore, novel findings of diagnostic and prognostic biomarkers related to CRC initiation and progression would be of great clinical relevance.

Long non-coding RNAs (lncRNAs) are a group of RNA transcripts, more than 200 nucleotides (nt) in length, with no or limited protein-coding capacity [4], which may play a role in diverse biological processes, such as cell cycle regulation, cell proliferation, differentiation, and apoptosis [5–7]. In addition, the aberrant expression of lncRNAs has been discovered in multiple tumors, where they function as oncogenes or tumor suppressor genes [8–11]. Among the cancer-related lncRNAs, one term ‘HOXA transcript at the distal tip’ (HOTTIP) has received increasing attention in recent years. The HOTTIP gene is located at the 5′ tip of the HOXA gene cluster in chromosome 7p15.2, which is an antisense non-coding transcript of 6838 nt in length. It was originally identified in anatomically distal human fibroblasts such as those from the hand, foot, or foreskin [12]. The role of lncRNA HOTTIP as an oncogene in varieties of human cancer including CRC has been extensively investigated [13–18]. It is significantly up-regulated in CRC tissue, which can regulate genes via epigenetic modification, and lncRNA–miRNA and lncRNA–protein interactions [12,19,20]. Therefore, lncRNA HOTTIP might serve as a candidate gene for cancerization and therapeutic targets of CRC.

Accumulating evidence has demonstrated that the expression and function of lncRNAs could be affected by single nucleotide polymorphisms (SNPs), the most common genetic variation in human genomes. They are universally present in lncRNA genes and proven to be of great value in cancer screening and therapy [21]. The association of HOTTIP SNPs with cancer susceptibility or prognosis has been preliminarily explored. For instance, patients with the HOTTIP rs5883064 C allele or rs1859168 A allele have an increased risk of lung cancer [22]. In addition, the rs1859168 A>C polymorphism may regulate HOTTIP expression and reduce the risk of pancreatic cancer in a Chinese population [23]. To date, however, no investigation has focused on the association between HOTTIP SNPs and CRC.

The present study explored the association of polymorphisms in the lncRNA HOTTIP gene with CRC risk and prognosis in a northern Chinese population. This work may provide clues for the identification of predictive biomarkers concerning CRC susceptibility and clinical outcomes, and thus also a theoretic basis for improving individualized diagnosis and therapy of CRC patients.

## Materials and methods

### Study participants

The project was approved by the Ethics Committee of the First Hospital of China Medical University. The research was carried out in accordance with the World Medical Association Declaration of Helsinki, and all subjects provided written informed consent. A total of 1848 individuals were enrolled in our study, including 884 cases and 964 controls. All the cases were recruited from histopathologically confirmed CRC patients attending the First Hospital of China Medical University, Shenyang, China, from September 2012 to March 2017. The control group consisted of healthy subjects seeking physical examination in the hospital and patients admitted to the Department of Anorectal Surgery with anal benign diseases diagnosed by digital rectal examination or other related methods during the same period. The controls were frequency-matched to CRC cases based on gender and age (±5 years), so that the frequency distribution of gender and age between the case and control groups had no remarkable statistical significance. A fasting venous blood sample (5 ml) was collected from each subject.

### Data collection

The epidemiological data of study participants including smoking, drinking, and *Helicobacter pylori* infection status were obtained from face-to-face inquiry or the medical records of inpatients. Clinicopathological data were collected from their pathological diagnosis reports. Clinical staging for CRC was determined according to the UICC/AJCC TNM staging system (2002) [24]. Regular follow-up was conducted for the CRC patients who underwent surgical treatment after an operation, which was completed by October 2017. All the cases were followed over a period of 6 months to 5 years. A total of 563 cases with available information of survival status and overall survival (OS) were involved in the prognosis study.

### SNP selection

**Figure 1:**
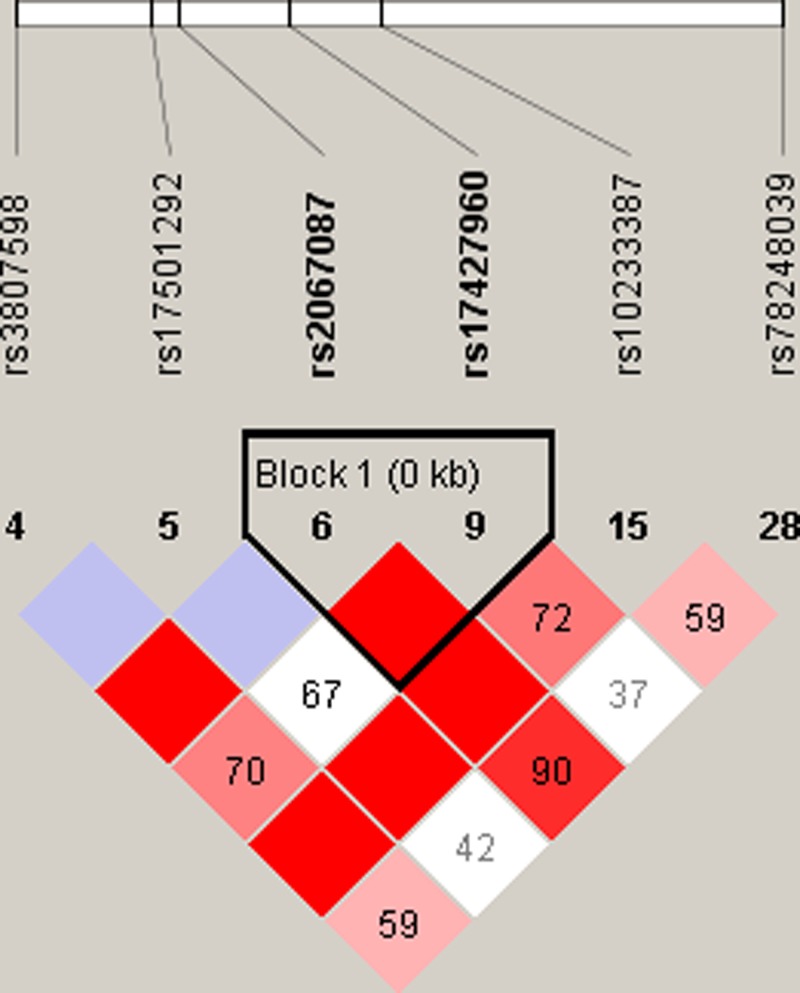
The LD plot of tagSNPs in the lncRNA HOTTIP gene At the top, the white strip and black lines represent the relative physical location and distance of all tagSNPs in the gene. The color shades of each block below represent the level of LD between any two SNPs (red: strongest LD; white: weakest LD). The numbers on the blocks are the *r*^2^ values of LD (percent).

First, we downloaded the sequences of the lncRNA HOTTIP gene using the 1000 Genomes Browser (https://www.ncbi.nlm.nih.gov/variation/tools/1000genomes/) after enlarging 2 kb of both upstream and downstream flanking sequences of the gene. The VCF to PED converter (http://www.internationalgenome.org/vcf-ped-converter) and Haploview 4.2 software were employed to select tagSNPs for HOTTIP. The selection criteria were: (i) the minor allele frequency (MAF) in CHB was more than 0.05; (ii) linkage disequilibrium (LD) *r*^2^ was less than 0.8; and (iii) the *P*-value for Hardy–Weinberg equilibrium (HWE) was more than 0.05. Consequently, five eligible tagSNPs were selected as research targets, including rs3807598, rs17501292, rs2067087, rs17427960, and rs78248039 (Figure 1). Potential function prediction was subsequently performed for all selected SNPs using the SNPinfo Web Server (https://snpinfo.niehs.nih.gov).

### SNP genotyping

DNA was extracted from a blood clot in each blood sample using a previously described method [25]. SNP genotyping was performed by Shanghai Baygene Biotechnology Company Limited (China) applying KASP (Kompetitive allele specific PCR) with an SNPLine platform (LGC Genomics, Hoddesdon, U.K.) [26]. For quality control, 10% of the samples were randomly selected for repeated detection and the duplicated samples reached 100% consistency.

### Statistical analysis

The chi-square test was used to evaluate the differences of host characteristics between case and control groups as well as HWE for studied SNPs in the control group. Multiple logistic regression was applied to estimate the association of each SNP with CRC risk and clinicopathological parameters in four genetic models by calculating the odds ratio (OR) with 95% confidence interval (95% CI) after adjusting for gender and age. The dominant and recessive models were respectively defined as variant homozygote + heterozygote vs. wild homozygote and variant homozygote vs. heterozygote + wild homozygote. Haplotype analysis was conducted using SHEsis online software (http://analysis.bio-x.cn/myAnalysis.php). Linear regression was employed to judge the cumulative effect of increasing risk genotypes. The interaction between each SNP and environmental factors on CRC risk was assessed using the log likelihood ratio test. We adopted the Kaplan–Meier method to calculate median survival time (MST) and mean survival time was provided when MST could not be determined. The log rank test was used to evaluate the differences in survival distribution between groups. Cox regression was applied to estimate the association of each SNP with the OS of CRC patients by calculating the hazard ratio (HR) with 95% CI both in univariate and multivariate models. All above-mentioned statistical analyses were conducted using SPSS 22.0 software (Chicago, IL, U.S.A.). The significance of all tests was two-sided and considered to be statistically significant when *P*<0.05. The Bonferroni correction was used to adjust *P*-values for multiple tests as needed.

## Results

### Characteristics of study participants

The study subjects included 884 CRC cases and 964 CRC-free controls. Although the two groups were roughly matched based on gender and age, the two variables were regarded as adjusted factors in subsequent analyses to eliminate their potential influence on study results. In addition, the infection rate of *H. pylori* in CRC patients was significantly higher than that in controls (*P*<0.001). No obvious difference was observed in the proportion of smokers or drinkers between the two groups (*P*>0.05, Supplementary Table S1).

### Association of HOTTIP SNPs with CRC risk

Among the five HOTTIP tagSNPs included in the study, the rs78248039 polymorphism failed in genotyping and was excluded from the final analysis. The genotype frequency of the other four SNPs in the control group all met HWE (*P*>0.05). The reference frequency of the healthy population (Beijing Han, China, NCBI database) is presented in Table 1.

**Table 1:** The association between HOTTIP SNPs and CRC risk^1^

SNP genotypes	NCBI Ref	CRC (%)	CON (%)	*P* (*P*_corr_)	OR (95% CI)
**rs3807598**		*n*=878	*n*=928		
CC	6 (13.6)	204 (23.2)	262 (28.2)		1 (Ref)
CG	24 (54.6)	420 (47.8)	457 (49.2)	0.132	1.19 (0.95–1.49)
GG	14 (31.8)	254 (28.9)	209 (22.5)	**0.001 (0.004)**	**1.57 (1.21–2.03)**
GG+CG vs. CC				**0.013 (0.052)**	**1.31 (1.06–1.62)**
GG vs. CG+CC				**0.002 (0.008)**	**1.40 (1.13–1.73)**
*P*_HWE_	0.395		0.718		
**rs17501292**		*n*=878	*n*=944		
TT	39 (90.7)	805 (91.7)	860 (91.1)		1 (Ref)
TG	4 (9.3)	71 (8.1)	82 (8.7)	0.613	0.92 (0.66–1.28)
GG	0 (0.0)	2 (0.2)	2 (0.2)	0.986	1.02 (0.14–7.28)
GG+TG vs. TT				0.621	0.92 (0.66–1.28)
GG vs. TG+TT				0.979	1.03 (0.14–7.34)
*P*_HWE_	0.749		0.975		
**rs2067087**		*n*=873	*n*=939		
GG	5 (11.1)	149 (17.1)	205 (21.8)		1 (Ref)
GC	18 (40.0)	398 (45.6)	470 (50.1)	0.207	1.18 (0.92–1.51)
CC	22 (48.9)	326 (37.3)	264 (28.1)	**<0.001 (<0.001)**	**1.70 (1.30–2.22)**
CC+GC vs. GG				**0.010 (0.040)**	**1.36 (1.08–1.72)**
CC vs. GC+GG				**<0.001 (<0.001)**	**1.52 (1.24–1.85)**
*P*_HWE_	0.654		0.877		
**rs17427960**		*n*=861	*n*=930		
CC	5 (11.4)	133 (15.4)	199 (21.4)		1 (Ref)
CA	16 (36.4)	400 (46.5)	450 (48.4)	**0.028 (0.112)**	**1.34 (1.03–1.73)**
AA	23 (52.2)	328 (38.1)	281 (30.2)	**<0.001 (<0.001)**	**1.74 (1.33–2.29)**
AA+CA vs. CC				**0.001 (0.004)**	**1.49 (1.17–1.90)**
AA vs. CA+CC				**0.001 (0.004)**	**1.41 (1.16–1.72)**
*P*_HWE_	0.401		0.452		

The results are in bold if *P*<0.05. Abbreviations: CON, control; NCBI Ref, reference frequency of the SNPs in healthy controls (Beijing Han, China, NCBI database); OR, odds ratio; *P*_corr_, *P*-values after Bonferroni correction; *P*_HWE_, HWE in control group. ^1^*P* was adjusted by gender and age.

The association between each SNP and CRC risk in overall subjects was estimated. Three SNPs including rs3807598, rs2067087, and rs17427960 were associated with increased CRC risk other than rs17501292. The variant types of rs3807598 (GG vs. CC: *P*=0.001, OR=1.57, 95% CI=1.21–2.03) and rs2067087 (CC vs. GG: *P*<0.001, OR=1.70, 95% CI=1.30–2.22) could, respectively, elevate the risk up to 1.57-fold and 1.70-fold when compared with their wild-types. The rs17427960 polymorphism conferred increased CRC risk in all genetic models, the highest ratio reaching 1.74-fold (CA vs. CC: *P*<0.001, OR=1.74, 95% CI=1.33–2.29, Table 1).

We further explored the association between each SNP and CRC risk stratified by host characteristics. Notably, rs3807598 and rs17427960 polymorphisms were found to elevate the risk in the subgroups of age ≤60 years and non-drinkers rather than the opposite subgroups. Rs2067087 and rs17427960 polymorphisms were linked to CRC risk only in the subjects without smoking or drinking history. Moreover, similar to the findings of the overall analysis, the rs17501292 polymorphism also showed no association with CRC risk (*P*>0.05, Supplementary Table S2).

### Cumulative effect of high risk HOTTIP SNPs

The cumulative effect of three SNPs related to CRC risk was then assessed. According to the results shown in Table 1, the best genetic models of each polymorphism were selected to identify their risk genotypes: GG vs. CG+CC for rs3807598; CC vs. GC+GG for rs2067087, and AA+CA vs. CC for rs17427960. All individuals were divided into four groups based on the number of risk genotypes they carried (0, 1, 2, and 3), and the significance of the linear trend was then analyzed. It appears that the susceptibility to CRC was significantly enhanced with the increasing number of SNP risk genotypes (*P*_trend_<0.001, Table 2).

**Table 2:** The cumulative effect of HOTTIP SNPs associated with CRC risk^1^

Number of SNP risk genotypes	CRC (%)	CON (%)	*P*	OR (95% CI)
	*n*=848	*n*=895		
0	130 (15.3)	193 (21.6)		1(Ref)
1	398 (46.9)	447 (49.9)	0.032	1.33 (1.03–1.73)
2	83 (9.8)	56 (6.3)	<0.001	2.23 (1.48–3.35)
3	237 (27.9)	199 (22.2)	<0.001	1.77 (1.32–2.37)
			***P*_trend_<0.001**	

The results are in bold if *P*_trend_<0.05. Abbreviation: CON, control.

^1^*P* was adjusted by gender and age.

### Haplotype analysis of HOTTIP SNPs for CRC risk

Haplotype analysis was performed to evaluate the association between haplotypes of the studied SNPs (rs3807598-rs17501292-rs2067087-rs17427960) and CRC risk. Four haplotypes were screened and three demonstrated significance. The C-T-C-A and G-T-C-A haplotypes contributed to elevated risk (*P*=0.047, OR=1.50, 95% CI=1.00–2.26; *P*<0.001, OR=1.27, 95% CI=1.10–1.45, respectively), while the G-T-G-C haplotype could reduce the risk (*P*<0.001, OR=0.73, 95% CI=0.64–0.84, Table 3).

**Table 3:** The association between haplotypes of HOTTIP SNPs and CRC risk^1^

Haplotypes	CRC (%)	CON (%)	*P*	OR (95% CI)^2^
C-G-C-A	52.76 (3.1)	47.54 (2.7)	0.425	1.18 (0.79–1.75)
C-T-C-A	58.07 (3.4)	41.21 (2.3)	**0.047**	**1.50 (1.00–2.26)**
C-T-G-C	617.75 (36.6)	778.38 (44.0)	**<0.001**	**0.73 (0.64–0.84)**
G-T-C-A	875.44 (51.9)	823.74 (46.6)	**<0.001**	**1.27 (1.10–1.45)**

The results are in bold if *P*<0.05. Abbreviation: CON, control. Haplotypes for ^1^rs3807598-rs17501292-rs2067087-rs17427960. ^2^The reference is the set of all the other haplotypes when one haplotype is regarded as an analyzed item.

### Interaction of HOTTIP SNPs with environmental factors

The effects of interaction between each SNP and environmental factors on CRC risk were also investigated, including smoking, drinking, and *H. pylori* infection status. Prior to this, their association with CRC risk was evaluated separately. It was shown that only *H. pylori* infection was significantly associated with CRC risk (smoking: *P*=0.231, OR=0.88, 95% CI=0.71–1.09; drinking: *P*=0.638, OR=0.94, 95% CI=0.72–1.22; *H. pylori* infection: *P*<0.001, OR=5.69, 95% CI=4.40–7.35); interaction analysis was subsequently performed. The AA+CA genotype of rs17427960 was found to negatively interact with drinking (*P*_interaction_=0.025, OR=0.45, 95% CI=0.22–0.91). No polymorphism was observed to interact with smoking or *H. pylori* infection (*P*>0.05, Table 4).

**Table 4:** The interaction effect between HOTTIP SNPs and environmental factors on CRC risk^1^

SNP genotypes	Smoking		Drinking		*H. pylori* infection	
	No	Yes	No	Yes	Negative	Positive
**rs3807598**	*n*=1044	*n*=491	*n*=1266	*n*=267	*n*=830	*n*=461
CG+CC						
Case/control	434/345	188/173	512/427	110/88	232/401	255/75
OR (95% CI)	1 (Ref)	0.86 (0.67–1.11)	1(Ref)	1.04 (0.77–1.42)	1 (Ref)	5.88 (4.34–7.97)
GG						
Case/control	171/94	79/51	212/115	38/31	85/112	105/26
OR (95% CI)	1.45 (1.08–1.93)	1.23 (0.84–1.80)	1.54 (1.18–2.00)	1.02 (0.63–1.67)	1.31 (0.95–1.82)	6.98 (4.41–11.04)
	*P*_interaction_=0.913		*P*_interaction_=0.148		*P*_interaction_=0.750	
**rs17501292**	*n*=1054	*n*=493	*n*=1276	*n*=269	*n*=841	*n*=463
GG+TG						
Case/control	48/43	24/21	58/55	14/9	33/51	22/3
OR (95% CI)	1 (Ref)	1.02 (0.50–2.10)	1 (Ref)	1.48 (0.59–3.68)	1 (Ref)	11.33 (3.14–40.90)
TT						
Case/control	557/406	243/205	665/498	135/111	285/472	337/101
OR (95% CI)	1.23 (0.80–1.89)	1.06 (0.68–1.67)	1.27 (0.86–1.86)	1.15 (0.74–1.80)	0.93 (0.59–1.48)	5.16 (3.16–8.43)
	*P*_interaction_=0.738		*P*_interaction_=0.368		*P*_interaction_=0.236	
**rs2067087**	*n*=1045	*n*=495	*n*=1269	*n*=269	*n*=832	*n*=462
GC+GG						
Case/control	375/326	171/160	445/399	101/84	200/372	227/76
OR (95% CI)	1 (Ref)	0.93 (0.72–1.21)	1 (Ref)	1.08 (0.78–1.48)	1 (Ref)	5.56 (4.07–7.59)
CC						
Case/control	224/120	97/67	273/152	48/36	113/147	132/27
OR (95% CI)	1.62 (1.24–2.12)	1.26 (0.89–1.78)	1.61 (1.27–2.05)	1.20 (0.76–1.88)	1.43 (1.06–1.93)	9.09 (5.81–14.24)
	*P*_interaction_=0.396		*P*_interaction_=0.200		*P*_interaction_=0.627	
**rs17427960**	*n*=1032	*n*=487	*n*=1259	*n*=258	*n*=826	*n*=453
CC						
Case/control	89/89	43/50	102/118	30/20	49/106	46/20
OR (95% CI)	1 (Ref)	0.86 (0.52–1.42)	1 (Ref)	1.74 (0.93–3.24)	1 (Ref)	4.98 (2.66–9.29)
AA+CA						
Case/control	503/351	220/174	610/429	113/95	264/407	306/81
OR (95% CI)	1.43 (1.04–1.98)	1.26 (0.89–1.80)	1.65 (1.23–2.20)	1.38 (0.94–2.01)	1.40 (0.97–2.04)	8.17 (5.38–12.41)
	*P*_interaction_=0.971		***P*_interaction_=0.025 (0.100)**^2^ **OR (95% CI)=0.45 (0.22–0.91)**		*P*_interaction_=0.671	

The results are in bold if *P*_interaction_<0.05. Abbreviation: CON, control. ^1^*P* for interaction was adjusted by gender and age. ^2^*P*-values after Bonferroni correction.

### Association of HOTTIP SNPs with CRC clinicopathological parameters and prognosis

As a series of important indicators related to CRC prognosis, common clinicopathological parameters were considered in the association study, including TNM stage, macroscopic type, histological type, depth of invasion, growth mode, and lymphatic metastasis. One polymorphism, rs3807598, was indicated to be associated with growth mode (CG vs. CC: *P*=0.026; GG+CG vs. CC: *P*=0.040, respectively, Supplementary Table S3).

Before the prognosis study, we estimated the potential effects of some host factors such as epidemiological and clinicopathological characteristics on the OS of CRC patients. It was shown that OS could be affected by TNM stage, macroscopic type, histological type, depth of invasion, growth mode, and lymphatic metastasis (*P*<0.001). As a result, these factors were regarded as adjusted variables in the multivariate Cox regression analysis (Table 5).

**Table 5:** The association between host factors and the OS of CRC patients

Factors	CRC patients	Death	MST (25%, 75%)	*P*	HR (95% CI)
Total	*n*=565	*n*=95			
Gender				0.815	
Male	384	63	47.0^1^ (NA, 44.0)		1 (Ref)
Female	181	32	47.6^1^ (NA, 46.0)		1.01 (0.65–1.56)
Age				0.101	
≤60	322	46	48.4^1^ (NA, 47.0)		1 (Ref)
>60	243	49	45.0^1^ (NA, 36.0)		1.27 (0.84–1.91)
Smoking				0.129	
Ever smoked	180	23	49.0^1^ (NA, 48.0)		1 (Ref)
Never smoked	383	72	46.4^1^ (NA, 43.0)		1.26 (0.78–2.02)
Drinking				0.176	
Drinker	107	14	49.6^1^ (NA, NA)		1 (Ref)
Non-drinker	456	81	46.6^1^ (NA, 43.0)		1.11 (0.62–1.97)
TNM stage				**<0.001**	
I+II	336	23	52.3^1^ (NA, NA)		1 (Ref)
III+IV	223	69	48 (NA, 24.0)		2.84 (0.38–21.31)
Macroscopic type				**<0.001**	
Protrude type	104	5	53.7^1^ (NA, NA)		1 (Ref)
Ulcerative/invasive type	458	90	45.6^1^ (NA, 38.0)		2.51 (0.97–6.48)
Histological type				**<0.001**	
High/middle differentiation	367	40	50.4^1^ (NA, NA)		1 (Ref)
Low differentiation	196	55	40.3^1^ (NA, 20.0)		2.14 (1.39–3.28)
Depth of invasion				**<0.001**	
T1+T2	114	6	53.6^1^ (NA, NA)		1 (Ref)
T3+T4	450	89	45.4^1^ (NA,36.0)		1.51 (0.62-3.68)
Growth mode				**<0.001**	
Nest	236	18	52.3^1^ (NA, NA)		1 (Ref)
Invasion	326	77	43.2^1^ (NA, 28.2)		2.28 (1.32–3.94)
Lymphatic metastasis				**<0.001**	
Positive	217	68	48 (NA, 24.0)		1 (Ref)
Negative	342	24	52.2^1^ (NA, NA)		0.87 (0.12–6.35)

The results are in bold if *P*<0.05. Abbreviations: MST, median survival time (months); NA, not available. ^1^Mean survival time was provided when MST could not be calculated.

Finally, the association between each SNP and the OS of CRC patients was explored. No association was found in the overall subjects (Supplementary Table S4), and thus stratified analysis was performed according to clinicopathological parameters. When the cases were grouped by macroscopic type, the heterozygote type TG of rs17501292 suggested better CRC prognosis in the ulcerative/invasive-type subgroup compared with the wild-type TT (*P*=0.043, HR=0.13, 95% CI=0.02–0.94, Table 6).

**Table 6:** The association between HOTTIP SNPs and CRC prognosis stratified by macroscopic type

SNP genotypes	CRC patients	Death	MST (M)	Univariate		Multivariate	
				*P*	HR (95% CI)	*P* (*P*_corr_)	HR (95% CI)
Protrude type							
**rs3807598**	*n*=104	*n*=5					
CC	21	1	51.9^1^		1(Ref)		1 (Ref)
CG	54	3	53.1^1^	0.772	0.72 (0.07–6.91)	0.677	1.64 (0.16–16.74)
GG	29	1	52.8^1^	0.957	0.96 (0.24–3.85)	0.041	NA
GG+CG vs. CC				0.796	0.75 (0.08–6.75)	0.918	1.12 (0.12–10.60)
GG vs. CG+CC				0.762	1.19 (0.40–3.56)	0.371	0.33 (0.03–3.71)
**rs17501292**	*n*=103	*n*=5					
TT	93	3	54.5^1^		1 (Ref)		1 (Ref)
TG	10	2	46.9^1^	0.082	0.20 (0.03–1.22)	0.188	3.93 (0.51–30.17)
**rs2067087**	*n*=102	*n*=5					
GG	15	0	NA		1 (Ref)		1 (Ref)
GC	48	2	54.4^1^	0.618	NA	0.784	NA
CC	39	3	49.9^1^	0.421	NA	0.956	NA
CC+GC vs. GG				0.501	NA	0.878	NA
CC vs. GC+GG				0.241	0.58 (0.24–1.44)	0.627	1.62 (0.23–11.20)
**rs17427960**	*n*=103	*n*=5					
CC	13	0	NA		1 (Ref)		1 (Ref)
CA	52	3	53.6^1^	0.562	NA	0.988	NA
AA	38	2	51.3^1^	0.506	NA	0.880	NA
AA+CA vs. CC				0.526	NA	0.894	NA
AA vs. CA+CC				0.777	0.88 (0.36–2.16)	0.662	0.64 (0.09–4.66)
Ulcerative/invasive type							
**rs3807598**	*n*=457	*n*=91					
CC	114	21	45.6^1^		1 (Ref)		1 (Ref)
CG	220	42	45.9^1^	0.910	0.97 (0.57–1.64)	0.273	1.35 (0.79–2.32)
GG	123	28	44.8^1^	0.606	0.93 (0.70–1.24)	0.123	1.61 (0.88–2.94)
GG+CG vs. CC				0.758	0.93 (0.57–1.51)	0.124	1.48 (0.90–2.45)
GG vs. CG+CC				0.585	0.94 (0.75–1.18)	0.190	1.37 (0.86–2.20)
**rs17501292**	*n*=456	*n*=91					
TT	425	90	45.0^1^		1 (Ref)		1 (Ref)
TG	31	1	51.2^1^	0.048	NA	**0.043 (0.172)**	**0.13 (0.02**–**0.94)**
**rs2067087**	*n*=453	*n*=92					
GG	84	15	46.0^1^		1 (Ref)		1 (Ref)
GC	212	44	44.8^1^	0.526	0.83 (0.46–1.49)	0.264	1.41 (0.77–2.57)
CC	157	33	45.5^1^	0.688	0.94 (0.69–1.28)	0.214	1.50 (0.79–2.82)
CC+GC vs. GG				0.555	0.85 (0.49–1.47)	0.194	1.45 (0.83–2.55)
CC vs. GC+GG				0.975	1.00 (0.81–1.25)	0.489	1.17 (0.75–1.83)
**rs17427960**	*n*=442	*n*=91					
CC	76	14	45.6^1^		1 (Ref)		1 (Ref)
CA	218	46	44.6^1^	0.587	0.85 (0.47–1.54)	0.350	1.34 (0.73–2.46)
AA	148	31	45.8^1^	0.890	0.98 (0.71–1.35)	0.594	1.19 (0.62–2.29)
AA+CA vs. CC				0.687	0.89 (0.50–1.57)	0.369	1.30 (0.73–2.32)
AA vs. CA+CC				0.708	1.04 (0.84–1.30)	0.824	0.95 (0.60–1.50)

The results are in bold if *P*<0.05. Abbreviations: MST(M), median survival time (months); NA, not available; *P*_corr_, *P*-values after Bonferroni correction. ^1^Mean survival time was provided when MST could not be calculated.

### Bioinformatics function prediction of HOTTIP SNPs

The basic information and function prediction results of HOTTIP tagSNPs are shown in Supplementary Table S5. Polymorphisms rs3807598, rs17501292, and rs2067087 belonged to the exon region of HOTTIP gene, and rs17427960 was the only intron locus. The rs2067087 polymorphism had both a relatively high RegPotention score and Converstion score. Furthermore, all studied SNPs were suggested to be related to transcription factor binding site (TFBS).

## Discussion

In the case–control study, we explored the association of four tagSNPs in the lncRNA HOTTIP gene with CRC risk and prognosis in a population of 1848 northern Chinese individuals. Rs3807598, rs2067087, and rs17427960 polymorphisms were newly found to be associated with CRC risk both in overall and stratified analysis. An obvious cumulative effect was demonstrated among them. Several haplotypes of HOTTIP SNPs were also shown to be associated with the risk. Additionally, the rs17427960 polymorphism had a two-way interaction with drinking. After adjustment of OS-related factors, the rs17501292 polymorphism indicated improvement of CRC prognosis in the ulcerative/invasive-type subgroup when the cases were categorized by macroscopic type. To the best of our knowledge, this is the first report of the association between lncRNA HOTTIP SNPs and CRC risk as well as prognosis.

The tumor-promoting effect of lncRNA HOTTIP in CRC has been demonstrated by many experiments. In 2015, Ren et al. [16] found that HOTTIP was highly expressed in CRC tissue. In 2016, it was suggested that HOTTIP could promote CRC growth partially via silencing of p21 expression [18]. Moreover, knockdown of the HOTTIP gene can inhibit CRC cell proliferation and migration and induce apoptosis by targeting SGK1 [17]. In view of this, the functional variants in HOTTIP are very likely to alter its expression and function, influence downstream molecules and pathways, and thus participate in the genesis and development of CRC. For the first time, our study found three HOTTIP tagSNPs could enhance CRC susceptibility. Among them, only rs3807598 has been reported to be involved in platinum-based chemotherapy toxicity in Chinese patients with lung cancer [27], while rs2067087 and rs17427960 have not been investigated yet. SNP function prediction showed that they all might affect TFBSs, which were DNA fragments that bind to transcription factors and were usually 5–20 bp in length. The alteration in TFBS caused by SNPs is very likely to change its spatial structure, and thus influence the smooth binding of transcription factors to gene promoters as well as transcriptional activity [28–30]. As the first step of gene expression, it will probably lead to a change of HOTTIP expression level. Both rs3807598 and rs2067087 were located in Exon 2 of the HOTTIP gene. The exon region could be completely reserved throughout the gene expression process [31]. Hence, they may result in the alteration of the secondary structure after transcription and thus the function of mature lncRNA. Furthermore, rs2067087 had a relatively high regulatory potential score and conservation score, suggesting it might be a highly conserved variation in the course of evolution with potential regulatory roles. Interestingly, the rs17427960 polymorphism is an intron locus with poor functional scores. In general, the intron sequences of a gene only influence the splicing during transcription and do not exist in a mature lncRNA [32]; however, it is still significantly associated with CRC risk. A possible explanation is that the observation regarding disease risk may not be due to the analyzed SNP rs17427960 but another unknown variant in strong LD harbored in HOTTIP or nearby genes [33]. In conclusion, HOTTIP rs3807598, rs2067087, and rs17427960 polymorphisms could be predictive biomarkers for CRC susceptibility. However, any hypothesis of a specific mechanism needs to be verified by further investigation.

Unlike other malignancies such as lung cancer, no single risk factor accounts for most CRC cases [34]. When the host characteristics and environmental factors were considered as stratification items, we found the three SNPs mentioned above were associated with increased CRC risk only in the subjects aged ≤60 years, non-smokers or non-drinkers, without significance in the opposite subgroups. It appears that the risk effect of HOTTIP SNPs could be modified by age, smoking, and drinking. Age is a well-known and important factor for CRC development [35]. The carcinogenic effect of cigarette smoking and excessive alcohol consumption has also been clarified by strong evidence, which contributes to increased incidence and mortality of CRC [36–39]. As a result, it is reasonable to believe that the association between HOTTIP SNPs and disease risk might be masked by these factors. Therefore, rs3807598, rs2067087, and rs17427960 polymorphisms may also be applied to predict CRC susceptibility in some specific populations, such as young people and individuals without smoking or drinking history. Furthermore, the role of one single factor on disease risk was limited and usually reported to be weak, but the combination of multiple factors may have stronger roles and markedly affect the susceptibility to cancer [40,41]. To identify some potential combination effects that cannot be revealed in univariate analyses, we evaluated the interactions between all the environmental factors and SNP genotypes, although only *H. pylori* infection was demonstrated to be associated with CRC risk. Notably, one of the studied SNPs, rs17427960, was shown to have an antagonistic effect with drinking and their interaction could reduce CRC risk to 0.45-fold, which indicated a potential modification of environmental factors on the biological function of HOTTIP polymorphisms in CRC risk. Previous studies have investigated the effect of the interaction between genetic variation and alcohol intake on CRC. For instance, drinkers with a (−/−) or (−/+) rs3830675 genotype in the *PTEN* gene have the highest CRC risk (OR=2.57) compared with the subjects that never consume alcohol [42]. However, no research has focused on the gene–environment interaction for HOTTIP SNPs and the mechanism involved in our findings needs to be further explored.

Due to the complicated factors participating in colorectal carcinogenesis, the capability of a single polymorphic site in risk identification is usually limited. It is well accepted that the combination of multiple SNPs has more advantages [43,44]. In our study, a significant dosage effect was observed in HOTTIP SNPs associated with CRC risk, suggesting the susceptibility to CRC could be enhanced by the increasing number of risk genotypes. CRC risk in individuals carrying the GG genotype of rs3807598, CC genotype of rs2067087, and AA+CA genotype of rs17427960 simultaneously could be elevated 1.77-fold, which is higher than any individual polymorphism. Furthermore, several haplotypes of rs3807598-rs17501292-rs2067087-rs17427960 were also found to add to the risk. Therefore, better diagnostic efficacy of CRC might be obtained by combining multi-variation in HOTTIP for detection.

The role of lncRNA HOTTIP exerted in the clinical outcome of CRC has been preliminarily investigated. The overexpression of HOTTIP in CRC tissue has been reported to be correlated with the prognosis of CRC patients [16]. Accordingly, it can be inferred that the functional variants in HOTTIP might be linked to CRC prognosis by affecting its expression. One studied SNP, rs17501292, was shown to be associated with improved OS of CRC cases. It is located in the exon region of the gene and might influence TFBSs. Considering its relatively high regulatory potential score, this polymorphism might be able to deliver a regulatory function. Interestingly, the protective effect of rs17501292 on CRC prognosis was only manifested in the tumor of ulcerative/invasive-type subgroup rather than overall subjects. The macroscopic type of cancer is an important pathological indicator and the malignant classification is closely related to poor patient survival. Hence, the significance of the rs17501292 polymorphism on CRC prognosis is likely to be covered by the risk effect of the malignant macroscopic type before stratification. However, further investigation is needed to elucidate the specific mechanism.

It should be noted that the present study has some limitations. First, the study is a retrospective case–control study, in which the design had its inherent limitations. Second, a small portion of the cases was lost to follow-up, which might influence the statistical power of prognosis analysis to some degree. Third, although our sample size is quite large, the study results need to be confirmed by future large-scale research, especially for rare genotypes. Additionally, we only focused on the association study between HOTTIP SNPs and CRC. In-depth functional experiments should be conducted to validate all assumptions regarding the mechanism involved.

## Conclusion

In summary, we designed a case–control study to investigate the association of lncRNA HOTTIP tagSNPs with CRC risk and prognosis. Three polymorphisms (rs3807598, rs2067087, and rs17427960) were associated with enhanced CRC risk both in overall and stratified analyses with an obvious cumulative effect among each other. A few haplotypes of rs3807598-rs17501292-rs2067087-rs17427960 were also associated with the risk. Furthermore, the rs17427960 polymorphism had a two-way interaction with drinking. One polymorphism, rs17501292, could improve the OS of CRC patients with a tumor of ulcerative/invasive type. These findings suggested HOTTIP SNPs could potentially be predictive biomarkers for CRC risk and prognosis. Our study provides clues for further exploration of novel lncRNA-based genetic biomarkers for the prediction of CRC susceptibility as well as prognosis, and thus possible access to individualized diagnosis and therapy of CRC patients.

## Supporting information

**Table S1.** The baseline characteristics of the subjects

**Table S2.** The association between HOTTIP SNPs and CRC risk stratified by host characteristics

**Table S3.** The association between HOTTIP SNPs and CRC clinicopathological parameters^a^

**Table S4.** The association between HOTTIP SNPs and CRC prognosis

**Table S5.** Function prediction results of HOTTIP SNPs

## Abbreviations

CRC: colorectal cancer
HOTTIP: HOXA transcript at the distal tip
HR: hazard ratio
HWE: Hardy–Weinberg equilibrium
LD: linkage disequilibrium
lncRNA: long non-coding RNA
MST: median survival time
nt: nucleotide
OR: odds ratio
OS: overall survival
SNP: single nucleotide polymorphism
TFBS: transcription factor binding site
95% CI: 95% confidence interval
