# Supplementary material for: Four novel polymorphisms in long non-coding RNA HOTTIP are associated with the risk and prognosis of colorectal cancer
Source: Biosci Rep. 2019 May 7;39(5):BSR20180573. doi: 10.1042/BSR20180573 (PMC6504661; doi:10.1042/BSR20180573)
Supplement: Supplementary file 1 [file bsr20180573_Supp1.pdf]

Table S1. The baseline characteristics of the subjects

Variables	CRC(%)	CON(%)
Total	n=884	n=964
Gender	*P*=0.724	
Male	580(65.6)	640(66.4)
Female	304(34.4)	324(33.6)
Age	***P*=0.032**	
Mean±SD	59.7±9.9	58.7±10.6
Median	60	58
Range	24-85	26-90
Smoking	n=878	n=690
	P=0.231	
Ever Smoker	269(30.6)	231(33.5)
Never Smoker	609(69.4)	459(66.5)
Drinking	n=878	n=688
	P=0.638	
Drinker	149(17.0)	123(17.9)
Non-drinker	729(83.0)	565(82.1)
H.pylori Infection	n=681	n=637
	***P*<0.001**	
Positive	362(53.2)	106(16.6)
Negative	319(46.8)	531(83.4)

Note: CRC, colorectal cancer; CON, control. The results are in bold if *P*<0.05.

Table S2. The association between HOTTIP SNPs and CRC risk stratified by host characteristics

Variables	SNP genotypes	CRC/CON	$P(P_{corr})$	OR(95%CI)
**rs3807598**		878/928		
Gender[a]				
Male	CC	139/175		1(Ref)
	CG	267/306	0.423	1.12(0.85-1.48)
	GG	169/141	**0.013(0.052)**	**1.50(1.09-2.06)**
	GG+CG vs. CC		0.104	1.24(0.96-1.61)
	GG vs. CG+CC		**0.014(0.056)**	**1.39(1.07-1.81)**
Female	CC	65/87		1(Ref)
	CG	153/151	0.147	1.34(0.90-1.98)
	GG	85/68	**0.032(0.128)**	**1.65(1.04-2.59)**
	GG+CG vs. CC		0.060	1.43(0.99-2.07)
	GG vs. CG+CC		0.107	1.36(0.94-1.96)
Age[a]				
≤60	CC	89/154		1(Ref)
	CG	229/268	**0.021(0.084)**	**1.45(1.06-1.99)**
	GG	133/119	**0.001(0.004)**	**1.87(1.30-2.68)**
	GG+CG vs. CC		**0.003(0.012)**	**1.58(1.17-2.13)**
	GG vs. CG+CC		**0.011(0.044)**	**1.45(1.09-1.94)**
>60	CC	115/108		1(Ref)
	CG	191/189	0.734	0.94(0.68-1.32)
	GG	121/90	0.363	1.20(0.81-1.76)
	GG+CG vs. CC		0.848	1.03(0.76-1.41)
	GG vs. CG+CC		0.154	1.26(0.92-1.73)
Smoking[b]				
Ever Smoker	CC	59/67		1(Ref)
	CG	129/106	0.141	1.39(0.90-2.16)
	GG	79/51	**0.027(0.108)**	**1.77(1.07-2.92)**
	GG+CG vs. CC		0.051	1.51(1.00-2.28)
	GG vs. CG+CC		0.102	1.41(0.93-2.13)
Never Smoker	CC	144/122		1(Ref)
	CG	290/223	0.454	1.12(0.83-1.52)
	GG	171/94	**0.013(0.052)**	**1.57(1.10-2.24)**
	GG+CG vs. CC		0.125	1.25(0.94-1.66)
	GG vs. CG+CC		**0.013(0.052)**	**1.44(1.08-1.93)**
Drinking[b]				
Drinker	CC	42/31		1(Ref)
	CG	68/57	0.663	0.87(0.48-1.60)
	GG	38/31	0.710	0.88(0.45-1.73)
	GG+CG vs. CC		0.632	0.87(0.50-1.53)
	GG vs. CG+CC		0.829	0.94(0.53-1.66)
Non-drinker	CC	161/157		1(Ref)

	CG	351/270	0.069	1.29(0.98-1.69)
	GG	212/115	**<0.001(0.001)**	**1.80(1.31-2.47)**
	GG+CG vs. CC		**0.005(0.020)**	**1.44(1.11-1.86)**
	GG vs. CG+CC		**0.002(0.008)**	**1.52(1.17-1.98)**
H.pylori Infection[b]				
Positive	CC	76/21		1(Ref)
	CG	179/54	0.970	1.01(0.56-1.81)
	GG	105/26	0.582	1.21(0.62-2.34)
	GG+CG vs. CC		0.828	1.06(0.61-1.85)
	GG vs. CG+CC		0.507	1.19(0.71-1.98)
Negative	CC	78/144		1(Ref)
	CG	154/257	0.557	1.11(0.79-1.56)
	GG	85/112	0.093	1.41(0.95-2.10)
	GG+CG vs. CC		0.276	1.20(0.87-1.65)
	GG vs. CG+CC		0.106	1.31(0.94-1.82)
**rs17501292**		878/944		
Gender[a]				
Male	TT	526/573		1(Ref)
	TG	49/56	0.752	0.94(0.63-1.40)
	GG	1/0	NA	NA
	GG+TG vs. TT		0.818	0.95(0.64-1.43)
	GG vs. TG+TT		NA	NA
Female	TT	279/287		1(Ref)
	TG	22/26	0.703	0.89(0.49-1.61)
	GG	1/2	0.589	0.51(0.05-5.82)
	GG+TG vs. TT		0.623	0.87(0.49-1.54)
	GG vs. TG+TT		0.594	0.52(0.05-5.84)
Age[a]				
≤60	TT	422/504		1(Ref)
	TG	27/43	0.295	0.77(0.46-1.26)
	GG	1/0	NA	NA
	GG+TG vs. TT		0.352	0.79(0.48-1.30)
	GG vs. TG+TT		NA	NA
>60	TT	383/356		1(Ref)
	TG	44/39	0.838	1.05(0.66-1.66)
	GG	1/2	0.621	0.54(0.05-6.12)
	GG+TG vs. TT		0.913	1.03(0.65-1.61)
	GG vs. TG+TT		0.611	0.53(0.05-5.99)
Smoking[b]				
Ever Smoker	TT	243/205		1(Ref)
	TG	24/21	0.793	0.92(0.49-1.72)
Never Smoker	TT	557/406		1(Ref)
	TG	46/41	0.338	0.81(0.52-1.26)
	GG	2/2	0.824	0.80(0.11-5.82)

	GG+TG vs. TT		0.327	0.80(0.52-1.24)
	GG vs. TG+TT		0.840	0.82(0.11-5.94)
Drinking[b]				
Drinker	TT	135/111		1(Ref)
	TG	14/9	0.865	1.08(0.45-2.62)
Non-drinker	TT	665/498		1(Ref)
	TG	56/53	0.212	0.78(0.52-1.15)
	GG	2/2	0.812	0.79(0.11-5.69)
	GG+TG vs. TT		0.206	0.78(0.53-1.15)
	GG vs. TG+TT		0.831	0.81(0.11-5.82)
H.pylori Infection[b]				
Positive	TT	337/101		1(Ref)
	TG	21/3	0.109	2.86(0.79-10.36)
	GG	1/0	NA	NA
	GG+TG vs. TT		0.099	2.93(0.82-10.56)
	GG vs. TG+TT		NA	NA
Negative	TT	285/472		1(Ref)
	TG	32/50	0.783	1.07(0.67-1.71)
	GG	1/1	0.920	1.15(0.07-18.76)
	GG+TG vs. TT		0.770	1.07(0.67-1.71)
	GG vs. TG+TT		0.914	1.17(0.07-18.89)
**rs2067087**		873/939		
Gender[a]				
Male	GG	105/134		1(Ref)
	GC	256/314	0.764	1.05(0.77-1.42)
	CC	215/179	**0.014(0.056)**	**1.51(1.09-2.09)**
	CC+GC vs. GG		0.180	1.22(0.91-1.62)
	CC vs. GC+GG		**0.002(0.008)**	**1.46(1.14-1.86)**
Female	GG	44/71		1(Ref)
	GC	142/156	0.095	1.46(0.94-2.26)
	CC	111/85	**0.002(0.008)**	**2.09(1.30-3.34)**
	CC+GC vs. GG		**0.016((0.064)**	**1.67(1.10-2.54)**
	CC vs. GC+GG		**0.008(0.032)**	**1.60(1.13-2.25)**
Age[a]				
≤60	GG	67/119		1(Ref)
	GC	212/284	0.142	1.30(0.92-1.84)
	CC	165/145	**0.001(0.004)**	**1.95(1.34-2.84)**
	CC+GC vs. GG		**0.014(0.056)**	**1.52(1.09-2.11)**
	CC vs. GC+GG		**<0.001(0.002)**	**1.62(1.24-2.13)**
>60	GG	82/86		1(Ref)
	GC	186/186	0.860	1.03(0.72-1.49)
	CC	161/119	0.130	1.35(0.92-2.00)
	CC+GC vs. GG		0.385	1.16(0.83-1.64)
	CC vs. GC+GG		**0.048(0.192)**	**1.34(1.00-1.80)**

Smoking[b]				
Ever Smoker	GG	45/50		1(Ref)
	GC	126/110	0.328	1.27(0.79-2.06)
	CC	97/67	0.091	1.56(0.93-2.61)
	CC+GC vs. GG		0.162	1.38(0.88-2.18)
	CC vs. GC+GG		0.162	1.32(0.90-1.93)
Never Smoker	GG	103/97		1(Ref)
	GC	272/229	0.390	1.16(0.83-1.62)
	CC	224/120	**0.002(0.008)**	**1.79(1.24-2.56)**
	CC+GC vs. GG		0.052	1.37(1.00-1.87)
	CC vs. GC+GG		**0.001(0.004)**	**1.61(1.23-2.11)**
Drinking[b]				
Drinker	GG	33/23		1(Ref)
	GC	68/61	0.299	0.70(0.36-1.37)
	CC	48/36	0.654	0.85(0.42-1.73)
	CC+GC vs. GG		0.366	0.75(0.40-1.40)
	CC vs. GC+GG		0.848	1.05(0.62-1.79)
Non-drinker	GG	115/123		1(Ref)
	GC	330/276	0.071	1.32(0.98-1.79)
	CC	273/152	**<0.001(<0.001)**	**1.93(1.39-2.66)**
	CC+GC vs. GG		**0.003(0.012)**	**1.53(1.15-2.03)**
	CC vs. GC+GG		**<0.001(<0.001)**	**1.59(1.25-2.02)**
H.pylori Infection[b]				
Positive	GG	55/20		1(Ref)
	GC	172/56	0.478	1.25(0.68-2.31)
	CC	132/27	0.057	1.93(0.98-3.78)
	CC+GC vs. GG		0.219	1.44(0.81-2.57)
	CC vs. GC+GG		**0.035(0.140)**	**1.71(1.04-2.82)**
Negative	GG	55/110		1(Ref)
	GC	145/262	0.575	1.12(0.76-1.64)
	CC	113/147	**0.034(0.136)**	**1.56(1.03-2.35)**
	CC+GC vs. GG		0.194	1.27(0.89-1.82)
	CC vs. GC+GG		**0.020(0.080)**	**1.43(1.06-1.93)**
**rs17427960**		861/930		
Gender[a]				
Male	CC	93/133		1(Ref)
	CA	255/299	0.206	1.23(0.89-1.68)
	AA	217/188	**0.003(0.012)**	**1.65(1.18-2.30)**
	AA+CA vs. CC		**0.029(0.116)**	**1.39(1.03-1.87)**
	AA vs. CA+CC		**0.004(0.016)**	**1.43(1.12-1.82)**
Female	CC	40/66		1(Ref)
	CA	145/151	0.050	1.57(1.00-2.48)
	AA	111/93	**0.005(0.020)**	**1.99(1.23-3.23)**
	AA+CA vs. CC		**0.013(0.052)**	**1.73(1.12-2.66)**

	AA vs. CA+CC		**0.040(0.160)**	**1.43(1.02-2.01)**
Age[a]				
≤60	CC	58/120		1(Ref)
	CA	212/265	**0.009(0.036)**	**1.62(1.13-2.33)**
	AA	171/158	**<0.001(<0.001)**	**2.18(1.49-3.20)**
	AA+CA vs. CC		**0.001(0.004)**	**1.83(1.29-2.58)**
	AA vs. CA+CC		**0.002(0.008)**	**1.54(1.18-2.02)**
>60	CC	75/79		1(Ref)
	CA	188/185	0.755	1.06(0.73-1.55)
	AA	157/123	0.184	1.31(0.88-1.96)
	AA+CA vs. CC		0.403	1.16(0.82-1.66)
	AA vs. CA+CC		0.109	1.27(0.95-1.70)
Smoking[b]				
Ever Smoker	CC	43/50		1(Ref)
	CA	124/104	0.158	1.43(0.87-2.33)
	AA	96/70	0.077	1.60(0.95-2.67)
	AA+CA vs. CC		0.090	1.49(0.94-2.36)
	AA vs. CA+CC		0.272	1.24(0.85-1.82)
Never Smoker	CC	89/89		1(Ref)
	CA	276/219	0.155	1.29(0.91-1.82)
	AA	227/132	**0.004(0.016)**	**1.73(1.19-2.50)**
	AA+CA vs. CC		**0.027(0.108)**	**1.45(1.04-2.01)**
	AA vs. CA+CC		**0.008(0.032)**	**1.43(1.10-1.87)**
Drinking[b]				
Drinker	CC	30/20		1(Ref)
	CA	65/57	0.244	0.66(0.33-1.33)
	AA	48/38	0.401	0.73(0.35-1.53)
	AA+CA vs. CC		0.246	0.68(0.35-1.31)
	AA vs. CA+CC		0.872	0.96(0.56-1.63)
Non-drinker	CC	102/118		1(Ref)
	CA	335/264	**0.009(0.036)**	**1.52(1.11-2.08)**
	AA	275/165	**<0.001(<0.001)**	**1.94(1.39-2.69)**
	AA+CA vs. CC		**0.001(0.004)**	**1.67(1.24-2.24)**
	AA vs. CA+CC		**0.003(0.012)**	**1.44(1.13-1.82)**
H.pylori Infection[b]				
Positive	CC	46/20		1(Ref)
	CA	167/50	0.164	1.57(0.83-2.95)
	AA	139/31	**0.031(0.124)**	**2.09(1.07-4.09)**
	AA+CA vs. CC		0.067	1.74(0.96-3.14)
	AA vs. CA+CC		0.082	1.54(0.95-2.49)
Negative	CC	49/106		1(Ref)
	CA	152/255	0.214	1.29(0.87-1.91)
	AA	112/152	**0.033(0.132)**	**1.58(1.04-2.41)**
	AA+CA vs. CC		0.084	1.39(0.96-2.02)

AA vs. CA+CC	0.078	1.31(0.97-1.77)

Note: [a], *P* was adjusted by the other factor of gender and age; [b], *P* was adjusted by gender and age; P_{corr}, *P* values after Bonferroni correction; CRC, colorectal cancer; CON, control; OR, odds ratio; CI, confidence interval; NA, not available. The results are in bold if $P<0.05$.

Table S3. The association between HOTTIP SNPs and CRC clinicopathological parameters[a]

SNP genotypes	TNM stage			Macroscopic type			Histological type			Depth of invasion			Growth mode			Lymphatic metastasis		
	Ⅰ+Ⅱ	Ⅲ+Ⅳ	*P*	Protrude type	Ulcerative/ Invasive type	*P*	High/Middle differentiation	Low differentiation	*P*	T1+T2	T3+T4	*P*	Nest	Invasion	P(P_{corr})	Positive	Negative	*P*
**rs3807598**	n=518	n=343		n=140	n=689		n=552	n=279		n=150	n=682		n=315	n=514		n=337	n=524	
CC	112(21.6)	91(26.5)		27(19.3)	165(23.9)		128(23.2)	65(23.3)		30(20.0)	163(23.9)		61(19.4)	132(25.7)		90(26.7)	113(21.6)	
CG	249(48.1)	164(47.8)	0.229	70(50.0)	331(48.0)	0.348	273(49.5)	129(46.2)	0.578	80(53.3)	322(47.2)	0.198	165(52.4)	236(45.9)	**0.026[b](0.104)**	160(47.5)	253(48.3)	0.179
GG	157(30.3)	88(25.7)	0.064	43(30.7)	193(28.0)	0.281	151(27.4)	85(30.5)	0.622	40(26.7)	197(28.9)	0.726	89(28.3)	146(28.4)	0.177	87(25.8)	158(30.2)	0.066
GG+CG vs. CC			0.107			0.301			0.920			0.319			**0.040[c](0.160)**			0.090
GG vs. CG+CC			0.139			0.490			0.347			0.595			0.965			0.171
**rs17501292**	n=520	n=341		n=139	n=690		n=552	n=279		n=150	n=682		n=314	n=515		n=335	n=526	
TT	480(92.3)	312(91.5)		126(90.6)	639(92.6)		509(92.2)	257(92.1)		141(94.0)	627(91.9)		286(91.1)	479(93.0)		308(91.9)	484(92.0)	
TG	39(7.5)	28(8.2)	0.712	13(9.4)	49(7.1)	0.303	41(7.4)	22(7.9)	0.830	9(6.0)	53(7.8)	0.470	28(8.9)	34(6.6)	0.229	26(7.8)	41(7.8)	0.983
GG	1(0.2)	1(0.3)	0.739	0(0.0)	2(0.3)	NA	2(0.4)	0(0.0)	NA	0(0.0)	2(0.3)	NA	0(0.0)	2(0.4)	NA	1(0.3)	1(0.2)	0.730
GG+TG vs. TT			0.683			0.374			0.970			0.407			0.319			0.980
GG vs. TG+TT			0.758			NA			NA			NA			NA			0.746
**rs2067087**	n=516	n=340		n=138	n=686		n=546	n=280		n=149	n=678		n=310	n=514		n=334	n=522	
GG	85(16.5)	64(18.8)		18(13.0)	124(18.1)		98(17.9)	45(16.1)		25(16.8)	118(17.4)		45(14.5)	98(19.1)		63(18.9)	86(16.5)	
GC	233(45.2)	160(47.1)	0.641	66(47.8)	313(45.6)	0.249	250(45.8)	129(46.1)	0.676	73(49.0)	307(45.3)	0.648	151(48.7)	228(44.4)	0.076	158(47.3)	235(45.0)	0.656
CC	198(38.4)	116(34.1)	0.224	54(39.1)	249(36.3)	0.223	198(36.3)	106(37.9)	0.484	51(34.2)	253(37.3)	0.785	114(36.8)	188(36.6)	0.199	113(33.8)	201(38.5)	0.199
CC+GC vs. GG			0.381			0.210			0.523			0.884			0.095			0.369
CC vs. GC+GG			0.201			0.513			0.642			0.483			0.951			0.163
**rs17427960**	n=508	n=337		n=138	n=676		n=541	n=275		n=150	n=667		n=308	n=506		n=331	n=514	
CC	81(15.9)	52(15.4)		14(10.1)	111(16.4)		90(16.6)	36(13.1)		22(14.7)	104(15.6)		45(14.6)	81(16.0)		52(15.7)	81(15.8)	
CA	229(45.1)	165(49.0)	0.575	70(50.7)	315(46.6)	0.085	253(46.8)	132(48.0)	0.259	78(52.0)	308(46.2)	0.488	150(48.7)	235(46.4)	0.483	163(49.2)	231(44.9)	0.654
AA	198(39.0)	120(35.6)	0.810	54(39.1)	250(37.0)	0.137	198(36.6)	107(38.9)	0.203	50(33.3)	255(38.2)	0.728	113(36.7)	190(37.5)	0.809	116(35.0)	202(39.3)	0.616

AA+CA vs. CC	0.831	0.091	0.191	0.811	0.601	0.985
AA vs. CA+CC	0.326	0.606	0.531	0.270	0.799	0.217

Note: [a], *P* was adjusted by gender and age; P_{corr}, *P* values after Bonferroni correction; [b], OR(95%CI)=0.66(0.46-0.95); [c], OR(95%CI)=0.70(0.50-0.98); OR, odds ratio; CI, confidence interval; NA, not available. The results are in bold if P<0.05.

Table S4. The association between HOTTIP SNPs and CRC prognosis

SNP genotypes	CRC patients	Death	MST(M)	Univariate		Multivariate	
				P	HR(95%CI)	*P*	HR(95%CI)
**rs3807598**	n=562	n=94					
CC	136	22	46.9[a]		1(Ref)		1(Ref)
CG	275	45	47.4[a]	0.903	0.97(0.58-1.61)	0.247	1.36(0.81-2.31)
GG	151	27	46.7[a]	0.671	0.94(0.71-1.25)	0.157	1.53(0.85-2.76)
GG+CG vs. CC				0.781	0.93(0.58-1.51)	0.131	1.46(0.89-2.38)
GG vs. CG+CC				0.672	0.95(0.76-1.19)	0.307	1.27(0.80-2.02)
**rs17501292**	n=560	n=94					
TT	519	91	46.8[a]		1(Ref)		1(Ref)
TG	41	3	50.7[a]	0.082	2.77(0.88-8.77)	0.080	0.35(0.11-1.13)
**rs2067087**	n=556	n=95					
GG	100	15	47.6[a]		1(Ref)		1(Ref)
GC	261	46	46.7[a]	0.486	0.81(0.45-1.46)	0.233	1.44(0.79-2.61)
CC	195	34	46.8[a]	0.531	0.91(0.67-1.23)	0.214	1.49(0.79-2.80)
CC+GC vs. GG				0.460	0.81(0.47-1.41)	0.163	1.49(0.85-2.61)
CC vs. GC+GG				0.858	0.98(0.80-1.21)	0.424	1.19(0.77-1.84)
**rs17427960**	n=546	n=94					
CC	90	14	47.3[a]		1(Ref)		1(Ref)
CA	271	49	46.4[a]	0.513	0.82(0.45-1.49)	0.278	1.40(0.76-2.56)
AA	185	31	47.4[a]	0.795	0.96(0.70-1.32)	0.579	1.20(0.63-2.29)
AA+CA vs. CC				0.593	0.86(0.49-1.51)	0.316	1.34(0.76-2.38)
AA vs. CA+CC				0.715	1.04(0.84-1.29)	0.759	0.93(0.60-1.45)

Note: CRC, colorectal cancer; MST(M), median survival time (months); [a], mean survival time was provided when MST could not be calculated; HR, hazard ratio; CI, confidence interval.

Table S5. Function prediction results of HOTTIP SNPs

SNP function predictions	**rs3807598**	**rs17501292**	**rs2067087**	**rs17427960**	**rs78248039**
Allele	C/G	T/G	G/C	C/A	A/T
Position	Exon	Exon	Exon	Intron	Exon
nsSNP	--	--	--	--	NA
Splicing site	--	--	--	--	NA
Splicing abolish domain	--	--	--	--	NA
Splicing ESE or ESS	--	--	--	--	NA
Stop Codon	--	--	--	--	NA
Polyphen	--	--	--	--	NA
SNPs3D (svm profile)	--	--	--	--	NA
SNPs3D (svm structure)	--	--	--	--	NA
TFBS	Y	Y	Y	Y	NA
miRNA (miRanda)	--	--	--	--	NA
miRNA (Sanger)	--	--	--	--	NA
RegPotential	0.0	0.265249	0.258321	0.0	NA
Conservation	0.007	0.000	0.990	0.000	NA

Note: nsSNP, nonsynonymous SNP; ESE, exon splicing enhancer; ESS, exon splicing silencer; TFBS, transcription factor binding site; NA, not available.
